# Gender, Family Histories, and Late-Life Economic Well-Being

**DOI:** 10.1093/geroni/igab046.927

**Published:** 2021-12-17

**Authors:** Deborah Carr, Pamela Smock, Teresa Ghilarducci

## Abstract

Text TK.

